# Breast implants following mastectomy in women with early-stage breast cancer: prevalence and impact on survival

**DOI:** 10.1186/bcr974

**Published:** 2004-12-23

**Authors:** Gem M Le, Cynthia D O'Malley, Sally L Glaser, Charles F Lynch, Janet L Stanford, Theresa HM Keegan, Dee W West

**Affiliations:** 1 Northern California Cancer Center, Fremont, California, USA; 2 Iowa Cancer Registry, University of Iowa, Iowa City, Iowa, USA; 3 Fred Hutchison Cancer Research Center, Division of Public Health Sciences, Seattle, Washington, USA

**Keywords:** breast implants, epidemiology, mastectomy, Surveillance, Epidemiology, and End Results, survival

## Abstract

**Background:**

Few studies have examined the effect of breast implants after mastectomy on long-term survival in breast cancer patients, despite growing public health concern over potential long-term adverse health effects.

**Methods:**

We analyzed data from the Surveillance, Epidemiology and End Results Breast Implant Surveillance Study conducted in San Francisco–Oakland, in Seattle–Puget Sound, and in Iowa. This population-based, retrospective cohort included women younger than 65 years when diagnosed with early or unstaged first primary breast cancer between 1983 and 1989, treated with mastectomy. The women were followed for a median of 12.4 years (*n *= 4968). Breast implant usage was validated by medical record review. Cox proportional hazards models were used to estimate hazard rate ratios for survival time until death due to breast cancer or other causes for women with and without breast implants, adjusted for relevant patient and tumor characteristics.

**Results:**

Twenty percent of cases received postmastectomy breast implants, with silicone gel-filled implants comprising the most common type. Patients with implants were younger and more likely to have *in situ *disease than patients not receiving implants. Risks of breast cancer mortality (hazard ratio, 0.54; 95% confidence interval, 0.43–0.67) and nonbreast cancer mortality (hazard ratio, 0.59; 95% confidence interval, 0.41–0.85) were lower in patients with implants than in those patients without implants, following adjustment for age and year of diagnosis, race/ethnicity, stage, tumor grade, histology, and radiation therapy. Implant type did not appear to influence long-term survival.

**Conclusions:**

In a large, population-representative sample, breast implants following mastectomy do not appear to confer any survival disadvantage following early-stage breast cancer in women younger than 65 years old.

## Introduction

Over the past 30 years, an estimated 1.5–2 million women have received breast implants in the United States [1]. Starting in the 1980s, widespread public health concern arose regarding their potential adverse health effects [2]. Numerous epidemiologic investigations have focused on systemic complications, particularly cancer and connective tissue disease, but have found no significantly increased short-term risk for these diseases [3,4].

Approximately 20% of breast implants are used for reconstruction in breast cancer patients following mastectomy [1]. In this population, however, the use of breast implants, while increasing, has not been well documented [5]. Furthermore, little research has addressed long-term survival, although the few studies conducted suggest that use of implants for breast reconstruction does not impact patient survival [6-9]. However, these studies were limited by comprising clinic-based samples not necessarily representative of all patients, by small sample sizes, by lack of information on type of implant, and by short durations of follow-up.

In 1993, the Surveillance, Epidemiology, and End Results (SEER) program of the National Cancer Institute implemented the Breast Implant Surveillance Study to document and validate postmastectomy breast implant usage in a population-based series of young, early-stage breast cancer cases. Since the SEER program also monitors patient vital status for life, survival up to 17 years exists in the study cohort. The purpose of the current analysis was to describe the use of breast implants and to examine the impact of breast implants on survival after breast cancer in this cohort.

## Methods

### Breast Implant Surveillance Study

The Breast Implant Surveillance Study was conducted during the period 1993–1994 in the United States. Patients were identified through the population-based SEER cancer registries in San Francisco–Oakland, CA, in Seattle–Puget Sound, WA, and in the state of Iowa. Eligible patients included the 5862 females diagnosed younger than age 65 with early-stage or unstaged first primary breast cancer in 1983, 1985, 1987, or 1989 and treated with mastectomy during their first course of therapy.

Participation involved completing a standardized questionnaire inquiring about implant status (right breast only, left breast only, both breasts, no implant, or unknown) and implant type (silicone gel, saline, double lumen [consisting of silicone gel and saline], other, and unknown type), and providing signed consent for release of medical records for validation of implant usage and implant type. Patients in the Seattle–Puget Sound region and the state of Iowa were mailed a self-administered questionnaire, and nonrespondents were contacted for a telephone interview. Women from the San Francisco–Oakland region were administered questionnaires by telephone.

Next-of-kin were asked about the deceased patient's implant status and for consent for medical record review. For women reporting breast implants, a medical record review of breast implant usage (including the date and type of implant received, and removal and replacement status) was conducted by trained abstractors for women who reported having a breast implant. Patient and tumor characteristics at diagnosis, including age, race/ethnicity, year, stage, histology, grade, radiation treatment, vital status, and cause of death, were obtained from the SEER database.

Although socioeconomic status (SES) is not routinely collected by SEER, we were able to assign census block group level measures of SES to a subset of subjects (1989 San Francisco Bay Area patients). Using data from the 1990 US Census, we examined the impact of living in census block groups characterized by low education, by poverty, and by occupation (median income, < 20% below poverty versus ≥ 20% below poverty; education, no high school diploma versus high school graduate; and occupation, blue collar versus nonblue collar [10]).

Patients who reported a breast implant were classified as having a particular type of implant if they had a unilateral implant, or if they had bilateral implants of the same type. Nineteen women with bilateral implants and discordant information about implant type were excluded from analyses stratified by the type of implant. An additional 133 women reporting implants but lacking implant information were excluded from all analyses requiring detailed implant information. The vital status (obtained annually through patient contact, death records, motor vehicle departments, voters' registration records, and Social Security files) was determined from the December 1999 SEER Public Use Tape. The outcome variables were death due to breast cancer and death due to nonbreast cancer causes, as routinely ascertained by SEER and as defined by the International Classification of Disease, Ninth Revision, codes 174.0–174.9 for deaths occurring between 1983 through 1998, and by the International Classification of Disease, 10th Revision, code C509 for deaths occurring in 1999. Survival time was calculated from the date of diagnosis to the earliest date: death, last known to be alive, or 31 December 1999 (study cutoff date).

### Statistical analysis

For descriptive analyses, women with breast implants were compared with those without implants on characteristics at diagnosis (SEER region of residence, age, year, race/ethnicity, marital status, stage, grade, and histology), using chi-square tests and Fisher's exact test to assess differences. Two-sided *P *< 0.05 was considered statistically significant.

Of the 5862 patients eligible for the study, 4968 (84.7% of those eligible) patients participated in the study. After restricting the sample to women with primary invasive breast cancer and known survival time, a final sample size of 4385 patients were used for all survival analyses. Survival estimates were computed using the Kaplan–Meier method, and differences in survival were compared using the two-sided log-rank test. To adjust for patient and tumor characteristics, the risks of nonbreast cancer mortality and death due to breast cancer were modeled using Cox proportional hazards regression after censoring deaths from other or unknown causes. The assumption of proportionality was tested and met for all covariates used in the Cox analysis. All analyses were performed using SAS Version 8.02 (SAS Institute, Inc., Cary, NC, USA).

## Results

### Study population

Eighty-five percent of study-eligible women completed the interview. Responders were slightly older compared with nonresponders. They also were more often non-Hispanic white, were diagnosed with *in situ *cancers, were less likely to have more than one primary tumor and were more likely to have lived until the end of the study period (Table 1).

Among the responders, 20% received a breast implant (Table 1). Their mean age at diagnosis was 47 years, and they were younger, on average, than women without implants. Somewhat higher proportions of women with implants were of non-Hispanic white race/ethnicity, resided in the San Francisco–Oakland region, were diagnosed in the late 1980s, and had tumors of lobular histology than women without implants. The percentage of women with *in situ *breast cancer was twice as high in women with breast implants as in nonimplanted women. In addition, women with implants were less likely to receive radiation therapy or to be diagnosed with more than one primary tumor.

### Implant characteristics and usage

Implant information was obtained for 866 women; these women were slightly older and more likely to be living than women with unknown implant information. Among the 1143 breast implants received (Table 2), the most common types were silicone gel and double lumen. The majority of women (67%) received a unilateral implant a median of 9.6 months after breast cancer diagnosis. Approximately one-third of the women had an implant removed; the majority of these women chose to have the implant replaced. Saline-filled implants were removed for 48% of women, although saline-filled tissue expanders (temporary implants) may be incorrectly included in this category. Fifty-four percent of women with 'other' implants had them removed, and 49% had them replaced.

### Survival

**Figure 1:**
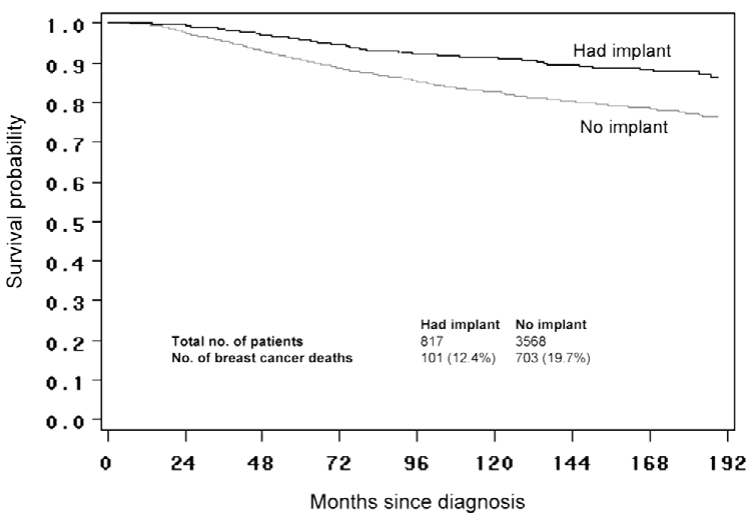
Age-adjusted breast cancer survival curve by breast implant status, 1983–1989.

At the end of the follow-up period, 231 (5.3%) patients did not have complete follow-up. Twenty-eight percent of all patients died, with nearly two-thirds of these deaths due to breast cancer (Table 3). Women with implants had a similar distribution of causes of death to those without implants (Table 3), except for a significantly larger proportion of deaths due to suicide – although this was based on a small number of deaths (0.4% versus 0.03% of all patients, respectively; *P *= 0.02). Among the 4385 patients with invasive breast cancer and known survival time, the 817 (19%) receiving a breast implant experienced better survival than women without implants, after adjustment for age at diagnosis (Fig. 1).

The multivariate Cox proportional hazards model showed that implant status was a significant factor associated with improved survival for deaths due to breast cancer and for nonbreast cancer mortality (Table 4). Risk of breast cancer death in women with implants was approximately one-half of that for women without implants, after adjustment for multiple clinical and sociodemographic factors. Age at diagnosis, stage, grade, histology, and radiation therapy were significant predictors of breast cancer death in this cohort, as they were for women without implants. With the exception of age, results were similar when modeled for nonbreast cancer mortality, although hazard rate ratios for women with implants were slightly higher overall. Among women with implants, the type of breast implant did not significantly impact survival, although women receiving saline implants had marginally lower risks than women receiving silicone gel implants.

For the subsample of 384 San Francisco Bay Area study subjects for whom we were able to assign census-level SES indicators, risk of death among women with breast implants compared with that among women without implants continued to be reduced (hazard ratio, 0.50; 95% confidence interval, 0.20–1.25) after adjustment for census block-group level SES variables.

## Discussion

In this large population-based study of breast cancer patients treated with mastectomy, risks of breast cancer death and nonbreast cancer mortality were lower in women with implants than in women without implants, after adjustment for potential confounders. Postmastectomy breast implants were used by one-fifth of patients who were slightly younger at diagnosis and were more likely to be of white race/ethnicity and to have *in situ *disease than women without implants. The silicone gel-filled implant was the most common type of implant received.

Although breast reconstruction has been shown to provide psychosocial benefits to breast cancer survivors [11-13], concerns have been raised that breast implants may increase the risk of local complications and systemic diseases, including certain cancers and autoimmune diseases [2,3,14,15]. Breast implants have been suggested to interfere with mammography, thereby facilitating delayed detection of breast tumors, and, consequently, decreased survival [16]. Despite recent Institute of Medicine recommendations to continue monitoring women with breast implants and to evaluate the potential long-term health effects [1], few research studies have addressed long-term health outcomes in this group. Moreover, these studies were often conducted on small, nonrepresentative samples without detailed information on implant type and history of use.

Georgiade and colleagues found that the survival time for 101 women undergoing breast reconstruction with breast implants was nonsignificantly better than that for 377 women without reconstruction, after adjustment for tumor grade, histology, lymph node involvement, and age at diagnosis, and after a median of 3 years of follow-up [6]. With a median of 13 years of follow-up, Petit and colleagues found that the risk of breast cancer death was marginally lower in 146 women who underwent breast reconstruction with silicone gel-filled implants than in a matched group without implants (relative risk, 0.6; 95% confidence interval, 0.3–1.1) [7]. Vandeweyer and colleagues compared 49 women who received saline-filled breast implants following mastectomy with a matched group of women who did not. They found no difference in the number of breast cancer deaths between the two groups [8]. In a matched analysis of 176 women with a mean of almost 6 years of follow-up, Park and colleagues found that women with breast implants after mastectomy had approximately a 70% reduced risk of death compared with women without implants (relative risk, 0.33; 95% confidence interval, 0.11–0.92) [9].

Our finding of better survival in women with breast implants is consistent with most of this research [6-8]. However, our study has the substantial advantages of being population-based, being large, having a long follow-up (median, 12.4 years), and including information on implant type and the implant removal and replacement. It is thus well suited to address public health concerns regarding the long-term survival and use of breast implants in women with early-stage, mastectomy-treated breast cancer. Such concerns have recently been re-evaluated in conjunction with the Food and Drug Administration hearings regarding the safety of silicone gel breast implants and their availability for the general market [17,18].

One explanation for our finding of reduced mortality in patients with breast implants may relate to self-selection rather than to a causal role of implants. Although most women who receive mastectomy are eligible to receive breast implants as part of breast reconstruction, surgeons may not recommend this surgery to women with health conditions such as obesity or a recent history of smoking that may contribute to postoperative complications, and may thus impact on survival [19,20]. In our data, the possibility of self-selection based on smoking is supported by the higher proportions of deaths from respiratory cancers and chronic obstructive pulmonary diseases in women without breast implants (Table 3). Further investigation is warranted for lifestyle factors (e.g. smoking, diet) and for comorbidities that may account for the survival advantage seen in women with breast implants.

In the present study, women with breast implants had a significant excess proportion of deaths due to suicide compared with women without implants. This finding, albeit based on small numbers, is consistent with observations from studies conducted in cosmetic breast implant patients [21,22] and suggests psychiatric consultation should also be considered for breast cancer patients seeking reconstructive surgery with breast implants. In any case, future studies with larger sample sizes are needed to confirm this finding in the breast reconstruction population.

An important bias of common concern in retrospective cohort studies is loss to follow-up. A total 231 (5.3%) of the 4385 patients included in the survival analysis did not have complete follow-up at the end of the study period. However, because of the relatively small percentage of patients lost to follow-up, we know that bias due to loss to follow-up has little impact on our survival findings since we found no substantial change in hazards ratios when we assumed the worst-case scenario that all patients lost to follow-up had all died or assumed that all patients lost to follow-up all lived until the end of the study period.

Furthermore, although our response rate was relatively high, differences between nonresponders and responders on several patient and tumor characteristics could have biased our findings. Although we were able to adjust for reported patient and tumor characteristics in our multivariate analyses, 356 women (nearly 40% of study-eligible patients) who did not participate in the study were deceased. In the unlikely event that all 356 deceased women had received breast implants, it is possible that the exclusion of these cases from our analysis could bias our results towards and beyond the null, and thereby overestimate the protective effects.

Although our survival analyses were adjusted for various demographic and clinical characteristics, our finding of better survival in women with breast implants could reflect uncontrolled confounding by social class, medical care, and psychological factors related to implant usage and survival. Among breast cancer patients treated with mastectomy, those choosing to have breast reconstruction have been shown to differ from women without breast reconstruction on SES, which may be an important factor affecting survival [23,24]. In a convenience sample of more than 200,000 breast cancer patients undergoing mastectomy between 1985 and 1995, Morrow and colleagues found that patients with a family income of $40,000 or more were twice as likely as patients with a family income of less than $40,000 to receive postmastectomy breast reconstruction [25]. Higher income may be a predictor of better survival after breast cancer, as women with higher incomes may have better access to cancer care and treatment. In the present study, SES did not alter the effect of implants on survival in the subset of women for whom SES measures were available. Differences in these area-level measures of SES are thus not likely to contribute substantially to the survival differences between breast cancer patients with and without implants in this study.

Additional unmeasured confounders related to the increased medical care of women with breast implants could explain the protective association of breast implants with cancer survival. Because women with breast implants may be more closely followed in their medical care, they may have recurrences diagnosed and treated earlier; thus they may experience better survival than women without implants. Although our study lacked information on breast cancer recurrence, we were able to examine the impact of subsequently diagnosed primary breast tumors. We observed that the proportion of women with two or more primary breast tumors was lower in women with breast implants than in women without (15% and 21%, respectively).

To address the possibility that a higher incidence of subsequently diagnosed primary breast tumors impacted survival in women without breast implants, we limited survival analyses to women with only one primary tumor (*n *= 3535) and found a consistently reduced risk of breast cancer death associated with breast implant usage (hazard ratio, 0.54; 95% confidence interval, 0.42–0.68), after adjusting for similar prognostic factors. Our findings also are consistent with results from studies showing a reduced risk of death in augmentation mammoplasty patients with at least 10 years of follow-up compared with the general population [26-29]. Furthermore, psychological factors underlying a woman's decision to obtain breast implants [30,31], including body image concerns and self-esteem, may play a role in lifestyle behaviors relevant to survival, although the extent to which they directly impact survival is unclear.

Several biological mechanisms have been proposed to explain how breast implants may influence survival outcomes [16,26,32,33]. Breast implants may stimulate a local immune response in which cancer cells are more likely to be destroyed [34]. Breast implants may compress breast tissue, reducing the flow of blood and thereby slowing the rate of cell or tumor growth. Breast implants may decrease the temperature of the breast by separating the breast tissue from the body, thereby decreasing the metabolic rate and slowing the growth rate of residual breast cancer cells [35]. These mechanisms may provide important clues in cancer prevention and warrant further investigation.

## Conclusions

With a median of more than 12 years of follow-up on patients, our population-based study shows that the risk of breast cancer mortality in patients with breast implants following mastectomy is about one-half of that for patients without implants, after adjustment for prognostic characteristics. Although patients in our study received implants in the 1980s and early 1990s, breast implants continue to be an integral part of breast reconstruction in breast cancer patients and have not changed dramatically in design. Thus, despite an overall decrease in implant use among breast cancer patients, breast implants remain an important and commonly used option for women considering reconstruction. Certainly, further research is needed to explain the survival differential in women with breast implants and those without, by examining potentially explanatory factors such as SES, comorbidity, smoking, or other lifestyle factors. However, based on this large, representative sample of breast cancer patients with extensive follow-up, we found that breast implants following mastectomy do not appear to confer any survival disadvantage following early-stage breast cancer in women younger than 65 years old.

## Abbreviations

SEER = Surveillance, Epidemiology, and End Results; SES = socioeconomic status.

## Competing interests

The author(s) declare that they have no competing interests.

## Authors' contributions

SLG, DWW, JLS, and CFL implemented the study and acquired the data. GML, SLG, and CDO participated in the design and conceptualization of the study. GML performed the statistical analysis and drafted the manuscript. GML, SLG, CDO, and THMK participated in the analysis and interpretation of the data. All authors read and approved the final manuscript.

## Figures and Tables

**Table 1:** Characteristics of mastectomy-treated breast cancer patients in the Surveillance, Epidemiology, and End Results (SEER) Breast Implant Surveillance Study, 1983–1989

Characteristic	All eligible patients (*n *= 5862)					Study responders only (*n *= 4968)				
	
	Nonresponders (*n *= 894)		Responders (*n *= 4968)		*P *value	Women with breast implants (*n *= 1018)		Women without breast implants (*n *= 3950)		*P *value
				
	*n*	%	*n*	%		*n*	%	*n*	%	
SEER region										
San Francisco–Oakland	468	52.4	1853	37.3	0.0001	416	40.9	1437	36.4	0.0001
Seattle–Puget Sound	190	21.3	1663	33.5		374	36.7	1078	27.3	
Iowa	236	26.4	1452	29.2		228	22.4	1435	36.3	
Year of diagnosis										
1983	246	27.5	1102	22.2	0.0001	176	17.3	926	23.4	0.0003
1985	241	27.0	1152	23.2		244	24.0	908	23.0	
1987	230	25.7	1405	28.3		319	31.3	1086	27.5	
1989	177	19.8	1309	26.3		279	27.4	1030	26.1	
Age at diagnosis										
< 35 years	59	6.6	175	3.5	0.0001	88	8.6	87	2.2	0.0001
35–44 years	231	25.8	919	18.4		334	32.8	585	14.8	
45–54 years	256	28.6	1545	31.1		370	36.4	1175	29.8	
55–64 years	348	38.9	2329	46.9		226	22.2	2103	53.2	
Mean age at diagnosis (years)	49.9	-	52.1	-	0.0001	46.7	-	53.4	-	0.0001
Race/ethnicity										
Non-Hispanic White	670	74.9	4444	89.5	0.0001	959	94.2	3485	88.2	0.0001
Non-Hispanic Black	78	8.7	150	3.0		13	1.3	137	3.5	
Hispanic	38	4.3	131	2.6		20	2.0	111	2.8	
Asian/Pacific Islander	86	9.6	179	3.6		16	1.6	163	4.1	
Other/unknown	22	2.5	64	1.3		10	1.0	54	1.4	
Stage at diagnosis										
*In situ*	78	8.7	567	11.4	0.04	199	19.6	368	9.3	0.0001
Localized	562	62.9	3141	63.2		601	59.0	2540	64.3	
Regional	241	27.0	1209	24.3		211	20.7	998	25.3	
Unstaged	13	1.5	51	1.0		7	0.7	44	1.1	
Histology										
Infiltrating ductal	687	76.9	3638	73.2	0.04	695	68.3	2943	74.5	0.0001
Lobular	44	4.9	364	7.3		106	10.4	258	6.5	
Infiltrating ductal and lobular	35	3.9	195	3.9		54	5.3	141	3.6	
Not otherwise specified/other	128	14.3	771	15.5		163	16.0	608	15.4	
Grade										
Well differentiated	17	1.9	150	3.0	0.02	29	2.9	121	3.1	0.91
Moderately differentiated	128	14.3	712	14.3		137	13.5	575	14.6	
Poorly differentiated	144	16.1	824	6.6		169	16.6	655	16.6	
Undifferentiated	17	1.9	185	3.7		39	3.8	146	3.7	
Unknown grade	588	65.8	3097	62.3		644	63.3	2453	62.1	
Number of primary tumors										
One primary only	771	86.2	4001	80.5	0.0001	867	85.2	3134	79.3	0.0001
Two or more primaries	123	13.8	967	19.5		151	14.8	816	20.7	
Radiation therapy during first course of treatment										
No	814	91.1	4626	93.1	0.03	969	95.2	3657	92.6	0.01
Yes	80	9.0	333	6.7		48	4.7	285	7.2	
Unknown	0	0	9	0.2		1	0.1	8	0.2	
Vital status^a^										
Alive	538	60.2	3696	74.4	0.0001	867	85.2	2828	71.6	0.0001
Dead	356	39.8	1272	25.6		151	14.8	1122	28.4	

^a^Final date of follow-up, December 1999.

**Table 2:** Characteristics of breast implants in mastectomy-treated breast cancer patients in the Surveillance, Epidemiology, and End Results Breast Implant Surveillance Study, 1983–1989 (*n *= 866)^a^

	Silicone gel (*n *= 333)		Saline (*n *= 149)		Double lumen (*n *= 314)		Other (*n *= 33)		Unknown (*n *= 37)		*P *value
	
	*n*	%	*n*	%	*n*	%	*n*	%	*n*	%	
Prevalence of type		38.4		17.2		36.3		3.8		4.3	
Implant removal status											
No known removal	244	73.3	77	51.7	216	64.9	15	45.5	25	67.6	0.0001
Removed	89	26.7	72	48.3	98	31.2	18	54.5	12	32.4	
Implant replacement status											
No known replacement	262	78.7	87	58.4	237	75.4	17	51.5	26	70.3	0.0001
Replaced	71	21.3	62	41.6	77	24.5	16	48.5	11	29.7	

^a^Excludes 133 patients with missing implant information and 19 patients with bilateral implants of disordant types

**Table 3:** Distribution of living and deceased patients by breast implant status in the Surveillance, Epidemiology, and End Results Breast Implant Surveillance Study, 1983–1989 (*n *= 4385)

	Women with breast implants (*n *= 817)		Women without breast implants (*n *= 3568)		Total (*n *= 4385)		*P *value^a^
		
	*n*	%	*n*	%	*n*	%	
Alive	676	82.3	2498	70.0	3174	72.4	< 0.0001
Deceased							
All malignant cancers							
Digestive system	2	0.2	32	0.9	34	0.8	0.08
Respiratory	3	0.4	51	1.4	54	1.2	0.01
Breast	101	12.4	703	19.7	804	18.3	< 0.0001
Female genital	4	0.5	20	0.6	24	0.5	0.80
Kidney	-	-	2	0.1	2	0.2	-
Brain	-	-	7	0.2	7	0.2	-
Melanoma	1	0.1	2	0.1	3	0.1	0.46
Multiple myeloma	-	-	3	0.1	3	0.1	-
Non-Hodgkin's lymphoma	-	-	3	0.1	3	0.1	-
Leukemia	2	0.2	2	0.1	4	0.1	0.16
Diabetes mellitus	2	0.2	13	0.4	15	0.3	0.60
Circulatory system							
Heart disease	4	0.5	65	1.8	69	1.6	0.01
Cerebrovascular disease	2	0.2	14	0.4	16	0.4	0.75
Atherosclerosis	-	-	3	0.1	3	0.1	-
Respiratory disease							
Pneumonia/influenza	1	0.1	10	0.3	11	0.3	-
Chronic obstructive pulmonary disease	2	0.2	23	0.6	25	0.6	0.21
Digestive system disease							
Stomach/duodenal ulcers	-	-	3	0.1	3	0.1	-
Chronic liver disease	1	0.1	7	0.2	8	0.2	-
Nephritis	-	-	7	0.2	7	0.2	-
All external causes							
Accidents	-	-	4	0.1	4	0.3	-
Suicides	3	0.4	1	0.03	4	0.3	0.02
Other cause of death	5	0.6	38	1.1	43	3.6	0.26
Unknown cause of death	7	0.9	32	0.9	39	3.2	0.91

Sum of deaths will not add to overall total since data are only shown for causes with at least two deaths in either group of women.

^a^*P *value for chi-square test or, where appropriate, Fisher's Exact Test.

**Table 4:** Proportional hazards regression model: hazard ratio and 95% confidence interval (CI) for mastectomy-treated breast cancer patients in the Surveillance, Epidemiology, and End Results (SEER) Breast Implant Surveillance Study, 1983–1989 (*n *= 4385)^a^

Covariate	Distribution of sample		Breast cancer mortality		Non-breast cancer mortality	
	
	*n*	%	Hazard ratio	95% confidence interval	Hazard ratio	95% confidence interval
Implant status						
No (referent)	3568	81.4	1.00	-	1.00	-
Yes	817	18.6	0.54	0.43–0.67	0.59	0.41–0.85
Age at diagnosis						
< 35 years	163	3.7	1.95	1.42–2.69	0.24	0.09–0.66
35–44 years	805	18.4	1.29	1.07–1.56	0.19	0.12–0.30
45–54 years	1332	30.4	1.02	0.86–1.20	0.35	0.26–0.46
55–64 years (referent)	2085	47.6	1.00	-	-	-
Race/ethnicity						
Non-Hispanic White (referent)	3915	89.3	1.00	-	1.00	-
Non-Hispanic Black	131	3.0	0.90	0.58–1.39	1.58	0.93–2.68
Hispanic	120	2.7	1.37	0.93–2.04	1.22	0.64–2.33
Non-Hispanic Asian	160	3.7	0.86	0.57–1.31	0.68	0.33–1.39
Other/unknown	59	1.4	0.75	0.37–1.50	1.36	0.60–3.07
Stage at diagnosis						
Local (referent)	3126	71.3	1.00	-	1.00	-
Regional	1208	27.6	2.28	1.97–2.63	1.86	1.65–2.09
Unstaged	51	1.2	3.80	2.41–5.99	3.35	2.28–4.92
SEER region						
San Francisco–Oakland (referent)	1624	37.0	1.00	-	1.00	-
Seattle–Puget Sound	1423	34.1	1.10	0.91–1.32	1.17	0.91–1.51
Iowa	1268	28.9	1.15	0.96–1.38	0.86	0.66–1.13
Year of diagnosis						
1983 (referent)	1037	23.7	1.00	-	1.00	-
1985	1003	22.9	1.06	0.88–1.29	0.96	0.73–1.27
1987	1232	28.1	0.97	0.80–1.18	1.05	0.78–1.40
1989	1113	25.4	0.84	0.68–1.04	1.12	0.80–1.56
Grade						
Well differentiated (referent)	142	3.2	1.00	-	1.00	-
Moderately differentiated	695	15.9	2.16	1.13–4.13	0.75	0.42–1.34
Poorly differentiated	817	18.6	3.45	1.82–6.53	0.79	0.44–1.40
Undifferentiated	182	4.2	4.97	2.52–9.78	1.23	0.60–2.52
Unknown	2549	58.1	2.41	1.28–4.52	0.85	0.50–1.45
Histology						
Ductal (referent)	3253	74.2	1.00	-	1.00	-
Lobular	279	6.4	1.09	0.82–1.44	1.26	0.88–1.81
Mixed ductal and lobular	167	3.8	1.06	0.74–1.53	0.66	0.32–1.33
Other	686	15.6	0.71	0.57–0.89	1.01	0.76–1.34
Radiation therapy						
No (referent)	4047	92.3	1.00	-	1.00	-
Yes	329	7.5	1.43	1.15–1.78	0.84	0.54–1.30
Unknown	9	0.2	4.68	1.74–12.60	-	-
Type of breast implant^b^						
Silicone gel (referent)	274	39.8	1.00	-	1.00	-
Saline	118	17.1	1.01	0.44–2.34	1.75	0.29–10.39
Double lumen	240	34.8	1.49	0.83–2.70	3.13	0.91–10.78
Other	26	3.8	1.11	0.24–5.03	-	-
Unknown	31	4.5	2.03	0.71–5.79	6.59	1.17–37.06

^a^Adjusted for age, year, stage at diagnosis, race/ethnicity, SEER region, tumor grade, histology, and radiation therapy.

^b^Model (*n *= 689) restricted to women with implant-specific information and concordant bilateral implants
